# Metabolic Reprogramming During Multidrug Resistance in Leukemias

**DOI:** 10.3389/fonc.2018.00090

**Published:** 2018-04-04

**Authors:** Raphael Silveira Vidal, Julia Quarti, Franklin D. Rumjanek, Vivian M. Rumjanek

**Affiliations:** ^1^Instituto de Bioquímica Médica Leopoldo de Meis, Universidade Federal do Rio de Janeiro, Rio de Janeiro, Brazil; ^2^Instituto de Nutrição Josué de Castro, Universidade Federal do Rio de Janeiro, Rio de Janeiro, Brazil

**Keywords:** multidrug resistance, glycolysis, glyceraldehyde-3-phosphate dehydrogenase, leukemia, reactive oxygen species

## Abstract

Cancer outcome has improved since introduction of target therapy. However, treatment success is still impaired by the same drug resistance mechanism of classical chemotherapy, known as multidrug resistance (MDR) phenotype. This phenotype promotes resistance to drugs with different structures and mechanism of action. Recent reports have shown that resistance acquisition is coupled to metabolic reprogramming. High-gene expression, increase of active transport, and conservation of redox status are one of the few examples that increase energy and substrate demands. It is not clear if the role of this metabolic shift in the MDR phenotype is related to its maintenance or to its induction. Apart from the nature of this relation, the metabolism may represent a new target to avoid or to block the mechanism that has been impairing treatment success. In this mini-review, we discuss the relation between metabolism and MDR resistance focusing on the multiple non-metabolic functions that enzymes of the glycolytic pathway are known to display, with emphasis with the diverse activities of glyceraldehyde-3-phosphate dehydrogenase.

## Background

Multidrug resistance (MDR) in cancer is the major factor impairing the success of conventional chemotherapy (1). Originally, many hopes were placed on the possibility that by inhibiting the activity of ABC transporters a reversal of the resistance would be attained. This was based on the knowledge that the transporters were shown to be capable of mediating the efflux of many chemotherapeutic drugs. Among the efflux transporters of the ABC superfamily, ABCB1 (P-gp), ABCC1 (MRP1), and ABCG2 (BCRP or MXR) have been described as major players in the development of MDR (2–4), with particular emphasis being given to ABCB1. However, it soon became apparent that inhibition of these transporters was not an effective approach since normal cells may also express these transporters and, therefore, inhibitors could sometimes generate an unacceptable toxicity. Furthermore, in experimental situations, where the down regulation of ABCB1 was achieved, a number of other effects were still observed. Now, it is quite clear that MDR is a multifactorial phenomenon that is involved in the regulation of survival and apoptosis, as well as a number of other cellular pathways. ABCB1 transporter expression, in cells with the MDR phenotype, is but one factor linked to pharmacological evasion of chemotherapeutic drugs (1, 5).

A case in point is the observation that hypoxia participates in the regulation of drug resistance. For instance, *ABCB1* gene expression and synthesis of functional proteins are induced by hypoxic environments (6). Furthermore, ABC transporters are expressed not only in MDR cancer cells, but also in a number of stem and progenitor cells. Additionally, it has been reported that hypoxia promotes an undifferentiated cell state in various stem and precursor cell populations, as well as in cancer stem cells (7–9). In this respect, it has also been suggested that NOTCH signaling is involved. However, it must be recalled that when cells are under hypoxic conditions, there is a metabolic shift from oxidative phosphorylation to glycolysis (10). This situation contrasts with cells under normoxia, in which glucose is first anaerobically catabolized to pyruvate which is then further catabolized along the Krebs cycle where NADH and FADH_2_ are reoxidized by the respiratory chain associated to the electron transport system. Incidentally, glycolysis is a hallmark in many types of tumor cells (11). This phenotype is in fact the basis of the so-called Warburg effect, also known as aerobic glycolysis. The Warburg effect describes a situation in which the glycolytic pathway is fully activated even in the presence of adequate oxygen supply (12). Although Warburg originally proposed that cancer was due to an impairment of mitochondrial function, it is accepted today that these organelles retain full oxidative capacities.

It must be mentioned, however, that apart from red blood cells, aerobic glycolysis is prevalent in highly proliferative cells, whether tumoral or not. Stem cells are a case in point (13). The common belief that cells undergoing glycolysis selected an inefficient form of energy production is misguided. Barring the comparative stoichiometry of ATP formation between glycolysis and OXPHOS, aerobic glycolysis is in fact an efficient form of ATP production due to the kinetic properties of the enzymes participating in the pathway which afford very fast fluxes compatible with the ATP demand of the rapidly growing cells. Beyond its role in bioenergetics, glycolysis constitutes a branch of the pentose phosphate pathway (PPP), since glucose-6-phosphate is also the substrate for glucose-6-phosphate dehydrogenase, the first enzyme of that pathway. Thus, glycolysis also contributes to the production of precursors for the biosynthesis of nucleotides (*via* generation of ribulose-5-phosphate). In addition, the PPP pathway promotes the formation of NADPH, an essential coenzyme for reductive biosynthetic processes such as that of fatty acids. NADPH also has an important role in maintaining the redox equilibrium. Similarly, glycolysis can be considered as an anaplerotic pathway by way of its participation in amino acids synthesis (*via* 3-phosphoglycerate or *via* pyruvate). Thus, from an energetic stand point glycolysis more than compensates the relatively small amounts of ATP produced when compared with oxidative phosphorylation.

However, it must be emphasized that tumors are in fact constituted by a mosaic of different cellular subpopulations. As such, from the biochemical point of view tumors can also be envisaged as being functionally heterogeneous. Accordingly, within the context of types of metabolism, tumors can be perceived as composed of subsets of resistant quiescent/slow-cycling cells that occasionally rely more on mitochondrial respiration and less on glycolysis. Likewise the same tumor could also harbor cells that are exclusively glycolytic (14, 15). Interestingly, the possibility of a switch that regulates mitochondrial function in the case of metastasis has been proposed. The results of Porporato et al. showed that overburdening the electron transport system may be an essential step in enhancing migration of cells *in vitro* and *in vivo* (16). The authors concluded that in order to achieve metastasis, mitochondria must be active, although not necessarily functional. By extension such findings suggest that in tumor cells there may be switches that constantly activate/inactivate mitochondrial function depending on changes dictated by the microenvironment that affect, for example, the availability of metabolites. The intermittent switching between anaerobic and oxidative metabolism seems to be a feature of metastasis. According to this scheme, accumulating data show that there is a tradeoff involving growth versus migration, i.e., cells which are proliferating prevalently exhibit a glycolytic type of metabolism, whereas migrating cells which proliferate less, rely more on mitochondria (16). Within this framework, it is known that the switch between the two main types of energy metabolism may be regulated by ATP demand. For example, cells expressing ABC transporters on their surfaces require a considerable amount of ATP in order to sustain the drug efflux activity. Additionally, it has been suggested that the transporter activity might suffer the impact of alterations in pH gradient due to the glycolytic phenotype (17). Normal cells maintain a gradient between acidic vesicular compartments and an alkaline cytoplasm a situation not observed in tumor cells that have an acidic cytoplasm. Nevertheless, it has been observed that MCF-7adr, that has an MDR phenotype, presents a similar pH gradient to that of normal cells (18, 19). This gradient contributes to the sequestration of drugs in acidic organelles and subsequent extrusion from the cell (19).

The mitochondrial electron transport chain generates reactive oxygen species (ROS) (20). In some instances, such as partial disintegration of complex I (21–23), oxidative stress may result from excessive production of ROS which is buffered by redox homeostasis. In turn, homeostasis is achieved by the participation of a number of enzymes such as catalase, superoxide dismutase, gluthatione peroxidase, and cofactors such as reduced gluthatione and NADPH. Control of redox equilibrium is important since an imbalance between the amount of ROS produced and of antioxidant systems may lead to DNA damage, particularly mitochondrial DNA, and other cell lesions.

Notwithstanding, the effect of ROS as agents of oxidative stress affecting the expression of ABCB1 is controversial. Both, downregulation and upregulation of the transporter have been reported (24, 25). Antioxidants (26), as well as products resulting from glycolysis can act as scavengers of free radicals. In this way, the energy metabolism could also play an indirect role as modulators of ABCB1 expression. For instance, it is known that under conditions leading to glycolysis inhibition, ABCB1 expression was observed to be downregulated. Conversely, when exogenous pyruvate was added to the tumor cells there was increased drug resistance and transporter expression (25).

Therefore, there is consensus that under normal conditions, glycolysis, and mitochondrial oxidative phosphorilation operate in concert in so far as energy production in the form of ATP is concerned.

Originally, glyceraldehyde-3-phosphate dehydrogenase (GAPDH) was thought to function exclusively as part of the glycolytic process in the cytoplasm, where its role is well established. However, under conditions of oxidative stress GAPDH may also redirect glyceraldehyde-3-P to glucose 6 phosphate (G6P) as a result from the reversal of part of the glycolytic reactions. The formed G6P then becomes the initial substrate of the PPP leading to the increase of NADPH production (27). GAPDH is particularly sensitive to H_2_O_2_-induced oxidation and it has been suggested that cytosolic GAPDH might function as a sensor for redox signals and an information hub to transduce these signals (28). Oxidative stress may also promote GAPDH aggregation leading to mitochondrial dysfunction and necrotic cell death *via* the permeability transition pore (29). Interestingly, under some circumstances GAPDH was shown to bind to band 3 protein, an anion transporter located on the inner side of the red blood cell membrane. In this membrane bound state, GAPDH along with other glycolytic enzymes contributed toward ATP channeling thus allowing its direct consumption by ion pumps without release into the cytoplasm (30). Such a role for GAPDH would be in keeping with an accessory function within the context of drug efflux. GAPDH, however, plays a number of other roles in different cell compartments, where several pools of GAPDH sense cellular stresses and activate cognate pathways to maintain homeostasis or activate cell death (31). It has also been reported that following exposure to stressors GAPDH translocate into the nucleus (32) where it may suffer ADP-ribosylation by NO (33). Apart from GAPDH, other enzymes of the glycolytic pathway are known to display multiple non-metabolic functions. In fact, of the 10 enzymes that constitute glycolysis, at least 7 have been shown to display extra-glycolytic activities that may bear on the MDR phenotype (34). For example, hexokinase II (HKII) binds to the mitochondrial voltage-gated anion channels and thus relieves the negative feedback effect of G6P (35). HKII overexpression is regulated by HIF-1α and also by c-Myc oncogene. HKII is also under the control of many miRNAs. When phosphohexoseisomerase, or phosphoglucoisomerase (PGI) is secreted by cells it acquires the status of a cytokine and is renamed as the autocrine motility factor (AMF). As such, AMF stimulates cell motility and so PGI is thought to be one of the factors driving metastasis (36). Besides, PGI is involved in many other activities such as apoptosis and EMT. Other glycolytic enzymes such as phosphofructokinase 1, aldolase and triose phosphate isomerase, phosphoglycerate mutase, and pyruvate kinase have all been reported to take part in several cellular functions that have a direct relation to tumorigenesis. Hence, it is entirely plausible that individually or collectively, the glycolytic enzymes and in particular GAPDH may constitute integral parts of the MDR phenotype by acting in a non-canonical fashion (37).

Alternatively, the metabolic rewiring of tumor cells may also result from gene rearrangement. Using yeast as a model system, genomic instability and the reprogramming of central metabolism have been approached (38). Regarding gene rearrangement, factors that normally contribute toward the integrity of the replicative process may be compromised. Drug resistance has been observed in yeast in which genome translocants were investigated (39). A parallel between yeast and tumor cells could thus be established even though the detailed mechanisms are still not understood. Indeed in MDR cells the over expression of mini chromosome maintenance 7 (MCM7) was detected (40).

Oxidative stress induces severe damage to proteins, lipids, and DNA. Normally, the generation of oxyradicals is prevented by the mitochondrial antioxidant system; however, the degree of damage will also depend on the repair capacity of the cell. DNA damage leads to the activation of Poly(ADP-ribose) polymerases or PARPs that are nuclear enzymes responsible for catalyzing the attachment of ADP-ribose units from nicotinamide adenine dinucleotide (NAD^+^) to acceptor proteins involved in the recognition and repair of DNA strands breaks. After repair is completed the Poly(ADP-ribose) (PAR) chains are degraded (41). During caspase mediated apoptosis PARP is cleaved into fragments that in turn inactivate the enzyme inhibiting repair to proceed (42, 43). It has been suggested that PARP inhibitors (PARPi) could be used in combination with chemotherapeutic drugs, but PARPi tends to be extruded from the cell by the ABCB1 transporter in MDR tumors (44).

Cell survival and energy metabolism in MDR tumor cells will be discussed in the next section.

## MDR, Chronic Myeloid Leukemia, and Energy Metabolism

Multidrug resistant cells may be very heterogeneous in their characteristics and survival pathways. In the present review, we will highlight, including our own data, some aspects related to the energy metabolism of two MDR chronic leukemia cell lines derived from the CML cell line, K562. The two cell lines were selected after exposure to different chemotherapeutic drugs. One cell line was exposed to vincristine, originating Lucena-1 (45), and the other exposed to daunorubicin, originating FEPS (46). Despite arising from the same parental cell, they were quite distinct. Comparative microarray analysis identified 130 differentially expressed genes between K562 versus Lucena-1, 1,932 between K562 versus FEPS, and 1,211 between Lucena-1 versus FEPS. *ABCB1* was overexpressed in both MDR cell lines, but highly overexpressed in FEPS, which is the most resistant line (47). Similarly, comparative proteomics of the parental cell line and the MDR counterparts indicated that K562 presented 560 unique proteins, Lucena-1 had 38 and FEPS 63 unique proteins. Lucena-1 and FEPS shared 929 proteins. From the latter, 112 were common only to Lucena-1 and FEPS (48). Results from another survey investigating the proteomic profiles of K562 and Lucena-1, identified 36 differentially expressed proteins between these two cell lines (40). From those, the leucine-rich PPR motif-containing protein and MCM7, as well as the expression of ABCB1 could be used as markers to identify patients that would respond or fail to therapy with the tirosine kinase inhibitor, Imatinib (40). The importance of ABCB1 in the resistance to Imatinib in the clinical setting has been demonstrated (49, 50), but it is not the only transporter involved. Tumor cells expressing ABCG2 are capable of Imatinib extrusion with great affinity (51, 52). In a different CML model, comparing the proteomic analysis of another MDR cell line derived from K562 selected with doxorubicin, Qinghong et al. highlighted 44 differentially expressed proteins. Some of the differentially expressed proteins were common to those observed in FEPS (53).

The possibility that overexpression of ABCB1 in these cell lines might reflect stem cell characteristics was analyzed by looking at the NOTCH pathway. Notch was overexpressed in FEPS, the most resistant cell line displaying the highest expression of *ABCB1*, and slow replication time (47). Another pathway present in both, normal stem cells and CML stem cells is the canonical Wnt pathway. This signaling pathway was shown to be more strongly activated to positively regulate ABCB1 in Lucena-1 cells when compared with the non-MDR K562. However, FEPS was not studied on this occasion (40). Conversely, both MDR cell lines had an increased expression of carbonic anhydrase and hemoglobin (47). This was confirmed by proteomics (48) suggesting that these cells are more differentiated compared with the parental erythroleukemic cell line K562 (54). Furthermore, they have the potential to maintain the intracellular pH (17). It has been reported that Imatinib induces erythrocytic differentiation in K562 cells and this is independent of blockade of apoptosis being also observed in resistant clones (55).

One characteristic of the two MDR cell lines described above (Lucena-1 and FEPS) is their antioxidant capacity. The elevated catalase activity observed in Lucena-1 provides these cells with protection against cytotoxic chemicals as well as UV radiation (56). Catalase activity is also elevated in FEPS and these cells also present increased Glucose 6 phosphate dehydrogenase (G6PD) activity (Vidal RS, Faria G, Maia RC, and Rumjanek VM, unpublished data). Despite the well-known role in redox homeostasis, G6PD is also involved in cell growth and signaling and this might be an equally important role in resistant cells (57). Many redox changes are now perceived to allow, in a localized compartment, a rapid and physiological signaling event that may regulate the activity of certain proteins (26, 58). Important players in regulating these intracellular effects are members of the thioredoxin family, including thioredoxin that has increased levels in Lucena-1 and FEPS (48), glutaredoxins, and peroxiredoxins. The third most upregulated gene in Lucena-1 when compared with K562 is *SESN3* that catalyzes peroxiredoxins leading to ROS detoxification (47). Furthermore, several members of peroxiredoxins, a family of antioxidant enzymes, are increased in FEPS (PRDX1, PRDX2, PRDX3, and PRDX6) or Lucena-1 (PDRX1) compared with K562 (47, 48). Therefore, ROS generated by the MDR tumor cell lines Lucena-1 and FEPS, are rapidly reduced. In this way, many anticancer drugs that act *via* generation of oxidative stress become ineffective.

**Figure 1:**
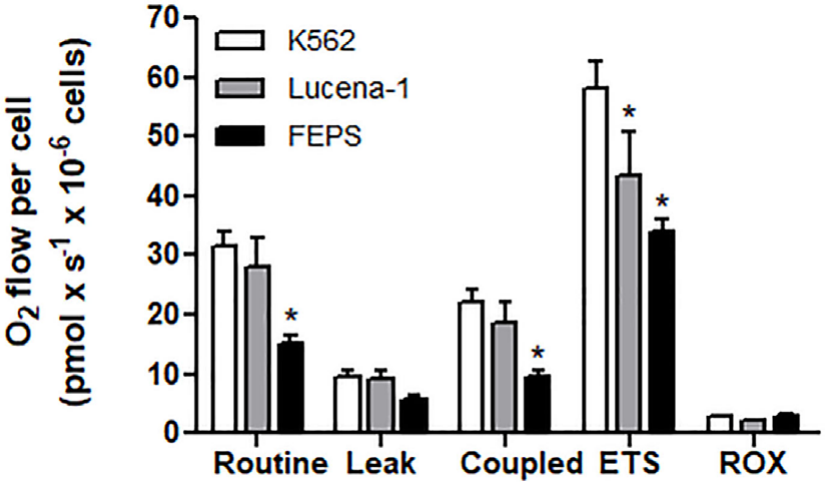
High-resolution respirometry of K562, Lucena-1, and FEPS cells. Respiratory parameters of intact K562, Lucena-1, and FEPS cells. Routine respiration—basal respiration of intact cells; leak respiration—rate of oxygen consumption after the addition of oligomycin, that is, uncoupled respiration; coupled respiration (routine–leak); ETS—maximum respiratory capacity (induced by the addition of FCCP); ROX—rate of oxygen consumption in the presence of rotenone and antimycin A, that is, respiration not associated with electron transport system. All the parameters were corrected by ROX values. The bars marked with asterisks denote the values that are significantly different with reference to K562 cells.

When the MDR cell lines and their parental counterpart, K562, were tested for oxygen consumption measured by high-resolution respirometry, the most resistant cell line, FEPS, reproducibly displayed comparatively lower values in all parameters measured (Figure 1). This result suggested that in FEPS, energy was probably being obtained *via* glycolysis. Gene expression (47) and protein expression (48) indicate an increase in pyruvate kinase levels in FEPS, whereas in Lucena-1 only increased protein expression was observed (48). Differences in the glycolytic pathway, with higher expression of hexoquinase 2, GAPDH and LDH, were observed using SKOV3 _TR_, an ovarian cell line transfected with the *ABCB1* gene (59).

Proteomic analysis of another MDR line obtained by doxorubicin selection using K562 as the parental cell, described upregulation of fructose-biphosphate aldolase A, fructose-biphosphate aldolase C, transaldolase, and alpha-enolase suggesting that the cells need more energy to survive chemical stress (53). FEPS cells, selected with daunorubicin also presented upregulated fructose-biphosphate aldolase A (48).

## GAPDH, MDR, and Cell Death

**Figure 2:**
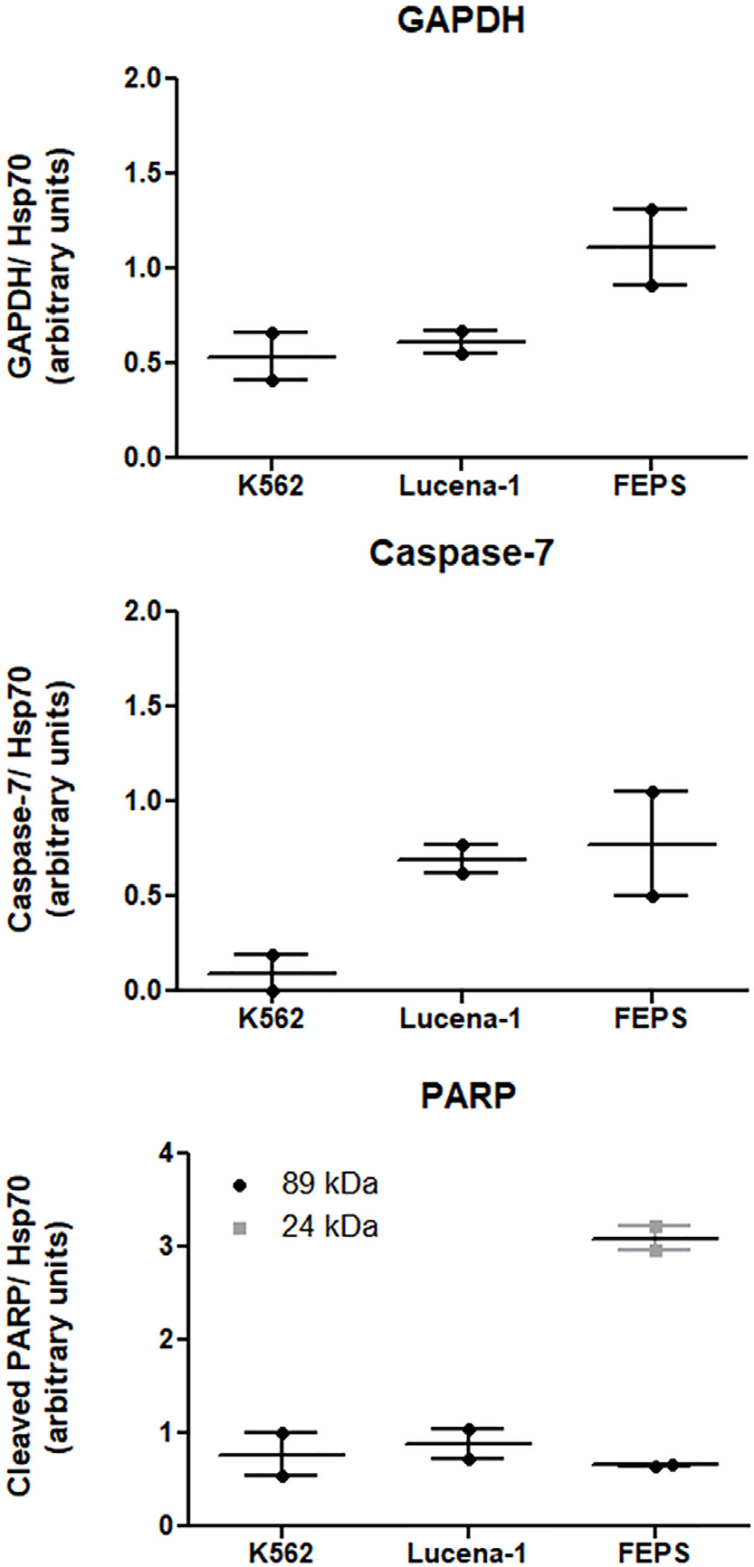
Expression of proteins related to cell death-resistance on K562, Lucena-1, and FEPS cells. Equal amounts of total cellular proteins (100 µg) were loaded in each lane for the detection of glyceraldehyde-3-phosphate dehydrogenase (GAPDH), pro-caspase-7, cleaved PARP, and Hsp70 (loading control) by western blot. Densitometric analysis of each lane was calculated using ImageJ Software. The data are expressed as arbitrary units and represent the mean of two independent experiments.

Among the differences observed in the present study was the variation found in the levels of GAPDH between K562 and its MDR counterparts. Using a proteomic approach (48), it was possible to verify that in relation to K562, the levels of GAPDH were 2.4 times higher in FEPS and 1.2 times in Lucena-1. These results were validated by western blot (Figure 2). Overexpression of GAPDH in Imatinib-resistant cells has been observed by other authors (60, 61). However, these results differ from those found by Cerezo et al. where no difference in GAPDH was observed in daunorubicin resistant cell lines overexpressing ABCB1 (62). Presumably the inconsistency reflects the well-known intrinsic differences encountered in tumor cells of different origin.

Interestingly, a recent report describes a strategy to circumvent the ABCB1 transporter activity by transferring constructs that specifically inhibited GAPDH into target tumor cells by using liposomes. Such treatment was effective *in vitro* and *in vivo* (63).

Therefore, the possibility exists that GAPDH plays a role in the resistance observed in some MDR tumors. Various non-glycolytic roles have been assigned to GAPDH (64). Post-translational modifications of GAPDH may dictate subcellular localization and different functions (31, 65). It has been proposed that Sirtuin1 expression retains GAPDH in the cytosol (66). However, no differences in gene or protein expression of Sirtuin 1, were observed in Lucena-1 or FEPS when compared with their parental cell line K562. As mentioned before, GAPDH translocates to the nucleus where it binds to DNA and participates in a number of DNA-dependent processes (67). However, the lack of differential expression of Sirtuin 1 in Lucena-1 and FEPS does not invalidate the hypothesis of GAPDH mediated action in the nucleus. GAPDH trafficking may occur by a number of mechanisms. For example, it is known that GAPDH can bind to microtubules and may thus get access to several intracellular organelles, including the nucleus (68).

It has been suggested that GAPDH is an inhibitor of caspase independent cell death (CID) (61, 69, 70). Death evasion to a number of chemotherapeutic drugs is the characteristic of cells displaying the MDR phenotype. Defects in the apoptotic process involving caspase activation to induce cell death have been observed in a number of multidrug resistant cell lines (62, 71).

Apoptosis is a result of caspase activation as a consequence of mitochondrial permeabilization and cytochrome c release. However, cells might also be killed following mitochondrial permeabilization even when caspase activation is inhibited, in the process known as CID. It has been suggested that, in such cases, cell death might occur as a result of a collapse of mitochondrial function, or the release of other proteins that could mediate the death process such as apoptosis-inducing factor (AIF), Smac/Diablo, HtrA2/Omi, Endonuclease G (72).

Mitochondrial permeabilization leading to cytochrome c release, and subsequent caspase activation, involves Bcl-2 family members having pro and anti-apoptotic properties. No difference in Bcl-2 levels could be detected when Lucena-1 and FEPS were compared with their parental non-MDR cell line K562 (46). On the other hand, the inhibitor of apoptosis survivin is increased in these cells, similarly to what has been described in K562/ADR (73) and other MDR cell lines (74, 75). Survivin suppresses cell death *via* caspase inhibition. Therefore, in a situation where caspases are inhibited, cell death could be a result of CID.

In a study, using CML cells, Lavallard et al. described that the tyrosine kinase inhibitor, Imatinib, was able to induce cell death in Bcr-Abl-positive cells by both caspase-dependent and independent manner (61). To induce CID, Imatinib was added to CML cells treated with caspase-inhibitors. In such situation cells transfected with GAPDH were protected from CID, however, these same transfected cells were not protected from Imatinib-induced apoptosis when no caspase-inhibitors were used. Furthermore, Imatinib-resistant K562 cells spontaneously overexpressed GAPDH compared with parental K562 and were protected from CID (61). This finding is in agreement with the results obtained with both Lucena-1 and FEPS MDR cell lines that also showed resistance to Imatinib and displayed higher levels of GAPDH compared with the parental K562 (Figure 2).

In experiments where caspase activity was inhibited but cytochrome c release occurred, it has been suggested that GAPDH acted by increasing the glycolytic metabolism and generating ATP as well as translocating to the nucleus where it is involved in the expression of Atg12 (70). Using GAPDH mutants, that either supported ATP production, but did not translocate to the nucleus or presented nuclear function albeit unable to produce ATP, these authors verified the requirement for the dual role played by GAPDH (70). The induction of Atg12 expression following mitochondrial permeabilization leads to autophagy with the removal of damaged mitochondria and subsequent cell survival (69).

Another death process involves the release of AIF from the mitochondrial intermembrane followed by its translocation to the nucleus. This step is a result of overactivation of the nuclear enzyme poly (ADP-ribose) synthetase 1 (PARP-1) (76). In the mitochondrial intermembrane space, AIF co-localizes with Hsp60. Due to its oxyredutase activity, AIF might act as a scanveger in the mitochondrial electron transport. However, AIF nuclear activity is independent of oxyredutase activity as mutations in the oxyredutase domain do not inhibit death-induction when it translocates to the nucleus (77). Once present in the nucleus AIF induces chromatin condensation and large-scale DNA fragmentation. The use of AIF mutants lacking the DNA-binding property abrogated cell death in spite of the preservation of AIF’s nuclear translocation (78).

In response to oxidative/nitrosative stress GAPDH binds to Siah and translocates to the nucleus where it activates PARP-1 (79). In the nucleus PARP-1 functions as a sensor to regulate celular DNA repair. PARP-1 overactivation, leading to AIF translocation to the nucleus, is a result of an attempt to restore damaged DNA. Usually, after repair is completed, the PAR chains are degraded. However, in cells with severely damaged DNA, nuclear PARP-1 is extensively activated and promotes the synthesis of an excess of PAR polymer. This reaches toxic levels and in the cytosol constitutes a death signal inducing AIF nuclear translocation (76, 80). The activation of PARP-1 results in the depletion of the cellular NAD^+^ and ATP pools. PARP-1 may be cleaved *in vitro* and *in vivo* by caspase-3 and caspase-7 originating two fragments an 89-kD catalytic fragment and a 24-kD DNA binding domain, capable of arresting the process (81). Similar fragments of 89 and 24 kD were detected in the MDR cell line FEPS, whereas only the 89 was observed in Lucena-1 cells (Figure 2). The relationship between PARP-1 activity and drug resistance is unclear but ABCB1 activity occurs at the expense of ATP. It has been described that cells from animals knockout for PARP-1 showed and increased ABCB1 expression and activity (82).

## Intracellular Calcium and MDR

The homeostatic control of cytosolic calcium concentration is of fundamental importance and changes in intracellular microenvironmental calcium levels can impact on cell survival, diverse cell functions, and cell death.

Proteins of the S100 family are calcium sensing proteins important in maintaining the homeostasis of the cell (83). Reports show that the calcium binding protein S100A6, also known as calcyclin or CACYBP, interacts with GAPDH (84). S100A6 is present in tumor cells as well as in normal fibroblasts, smooth, and heart muscle cells (83). This protein, as well as its gene expression, is increased twofold in FEPS compared with K562 (48). However, it is not clear how the interaction of S100A6 and GAPDH might play a role inhibiting CID.

Sorcin is another calcium–protein associated with MDR (85–87). Using K562 cells overexpressing sorcin, it was observed that ABCB1 was upregulated. The opposite was also true. In MDR-K562 cells selected by exposure to doxorubicin it was found that sorcin was upregulated, suggesting that ABCB1 and sorcin may regulate the expression of each other (85). When sorcin was analyzed using the MDR cell line Lucena-1 selected by vincristine, an increased gene and protein expression was also observed compared with the parental K562 (47, 48). This result is in agreement with the proteomics of another doxorubicin-induced K562 where the protein sorcin was also increased (53).

Despite the fact that the relationship between sorcin and ABCB1 has been known for a number of years (88), the meaning of such relation is still unknown. Differences in intracellular calcium levels have been reported in cells presenting the MDR phenotype related to ABCB1 overexpression (89). Some of these differences could be attributed to increased sorcin levels but in most cases, a causal relationship was not established.

Cells transfected with cDNA for *ABCB1* did not mobilize calcium when the SERCA inhibitor thapsigargin was used (90). The explanation proposed by Gutheil et al. was that thapsigargin was being extruded. However, no calcium mobilization could be induced in Lucena-1 cells using thapsigargin in a condition in which the inhibitor could not be extruded from the cell, suggesting that drug efflux could not fully explain the lack of mobilization (91). Considering that the main intracellular calcium store is the endoplasmic reticulum (ER) and that thapsigargin elicits ER-stress inhibiting the ER calcium pump, this is another death-inducing mechanism that is altered in MDR cells. Similarly, other workers using two types of resistant cells, one selected with vincristine and one with a stable transfection with a gene encoding ABCB1, also described the reduced sensitivity of MDR cells to thapsigargin (92, 93). Moreover, using immunofluorescence, they observed differences in the localization of the ER proteins ryanodine receptor (RyR), inositol 1,4,5-trisphosphate receptor and calnexin, between the MDR cells and their parental (92). Despite the fact that no statistical difference was observed in the amount of GAPDH comparing the two types of resistant cells with the parental one, treatment with thapsigargin decreased the protein content of GAPDH as well as ABCB1, suggesting a relationship between the two (93).

## Conclusion

The MDR phenotype is a very complex phenomenon. There is now a growing awareness that drug resistance transcends the ABCB1 transporters and involves other elements associated to metabolic reprogramming. Accordingly, the enzymes of the glycolytic pathway have been shown to exert several regulatory roles that bear on drug resistance. Therefore, pharmacological interference studies on the MDR phenotype may have a better chance to succeed if they are expanded by include as potential targets GAPDH and other enzymes of the glycolytic pathway. In this review, many ancillary roles of such enzymes have been commented and underlined those that affect, for instance, the supply of ATP for drug extrusion purposes. Furthermore, attention was called to the fact that cells displaying the MDR phenotype also displayed enhanced expression/activity of enzymes involved in the maintenance of redox homeostasis of tumor cells. Thus, chemotherapy aiming at the combined abrogation of drug resistance and careful modulation of the redox equilibrium may open up alternative avenues for the control of tumor growth and metastasis.

## Author Contributions

VR wrote and reviewed the article. RV and JQ contributed to the writing and data gathering. JQ contributed to the editing and submission of this manuscript. FR contributed to the writing and reviewed the article.

## Conflict of Interest Statement

The authors declare that the research was conducted in the absence of any commercial or financial relationships that could be construed as a potential conflict of interest. The reviewer CVB and the handling Editor declared their past shared affiliation.
