# Corrigendum: Metabolic Reprogramming During Multidrug Resistance in Leukemias

**DOI:** 10.3389/fonc.2018.00441

**Published:** 2018-11-12

**Authors:** Raphael Silveira Vidal, Julia Quarti, Mariana Figueiredo Rodrigues, Franklin D. Rumjanek, Vivian M. Rumjanek

**Affiliations:** ^1^Instituto de Bioquímica Médica Leopoldo de Meis, Universidade Federal do Rio de Janeiro, Rio de Janeiro, Brazil; ^2^Instituto de Nutrição Josué de Castro, Universidade Federal do Rio de Janeiro, Rio de Janeiro, Brazil

**Keywords:** multidrug resistance, glycolysis, glyceraldehyde-3-phosphate dehydrogenase, leukemia, reactive oxygen species

Mariana Figueiredo Rodrigues was not included as an author in the published article. The authors apologize for this error and state that this does not change the scientific conclusions of the article in any way. The original article has been updated.

## Conflict of interest statement

The authors declare that the research was conducted in the absence of any commercial or financial relationships that could be construed as a potential conflict of interest.

